# MRI for discriminating metastatic ovarian tumors from primary epithelial ovarian cancers

**DOI:** 10.1186/s13048-015-0188-5

**Published:** 2015-08-28

**Authors:** Yanhong Xu, Jia Yang, Zaixian Zhang, Guixiang Zhang

**Affiliations:** Department of Radiology, Shanghai First People’s Hospital, Shanghai Jiaotong University School of Medicine, 100 Haining Road, Shanghai, 200080 People’s Republic of China

**Keywords:** Ovary, Primary epithelial ovarian cancer, Metastatic, Magnetic resonance imaging

## Abstract

**Aims:**

To find specific magnetic resonance imaging (MRI) features to differentiate metastatic ovarian tumors from primary epithelial ovarian cancers.

**Methods:**

Eleven cases with metastatic ovarian tumors and 26 cases with primary malignant epithelial ovarian cancers were retrospectively studied. All features such as patient characteristics, MRI findings and biomarkers were evaluated. The differences including laterality, configuration, uniformity of locules, diffusion weighted imaging (DWI) signal of solid components and enhancement of solid portions between metastatic ovarian tumors and primary epithelial ovarian cancers were compared by Fisher’s exact test. Median age of patients, the maximum diameter of lesions and biomarkers were compared by the Mann-Whitney test.

**Results:**

Patients with metastatic ovarian tumors were younger than patients with primary epithelial ovarian cancers in the median age (*P* = 0.015). Patients with bilateral tumors in metastatic ovarian tumors were more than those of primary epithelial ovarian cancers (*P* = 0.032). The maximum diameter of lesions in metastatic ovarian tumors was smaller than that of primary epithelial ovarian cancers (*P* = 0.005). The locules in metastatic ovarian tumors were more uniform than those of primary epithelial ovarian cancers (*P* = 0.024). The enhancement of solid portions in metastatic ovarian tumors showed more moderate than that of primary epithelial ovarian cancers (*P* = 0.037). There was no statistically significant difference between the two groups in configuration, DWI signal of solid components and ascites. Biomarkers such as CA125 and human epididymis protein 4 (HE4) in metastatic ovarian tumors showed less elevated than that of primary epithelial ovarian cancers.

**Conclusions:**

Significant differences between metastatic ovarian tumors and primary epithelial ovarian cancers were found in the median age of patients, laterality, the maximum diameter of lesions, uniformity of locules, enhancement patterns of solid portions and biomarkers. Metastatic ovarian tumors usually presented in the younger patients, smaller-sized, more bilateral lesions, more uniform of locules, more moderate enhancement of solid portions, and less elevated levels of CA125 and HE4 than those of primary epithelial ovarian cancers.

## Background

The optimal management and prognosis of metastatic ovarian tumors depend on the origin of the primary tumor [1, 2]. For primary ovarian cancers, management will be based on cytoreductive surgery and systemic therapy (depending on stage). Therefore, preoperative discrimination is very critical. However, by now, it is difficult to discriminate these tumors by imaging, or even by histopathology in some cases, as macroscopic and microscopic features of metastatic ovarian tumors and primary epithelial ovarian cancers are often similar. Therefore, they cannot be definitively classified without further clinical evaluations [3, 4]. Histological preoperative diagnosis is now and then impossible because there is a risk of dissemination of primary ovarian cancer on an early stage otherwise. In general, the therapy methods and prognoses of metastatic ovarian tumors are different from those of primary epithelial ovarian cancers [5]. Therefore, discriminations between them are very critical. However, there is a lack of comprehensive imaging studies concerning the distinctions. Magnetic resonance imaging (MRI) is a useful tool for investigation and description of characteristic signs for the preoperative diagnosis of an ovarian lesion [1]. The object of this study is to detect specific MRI features of metastatic ovarian tumors that can be discriminated from primary epithelial ovarian cancers.

## Methods

### Study subjects

This retrospective study was approved by the institutional review boards of Shanghai First People’s Hospital, Shanghai, China. The informed consent requirement was waived. We searched for data of patients with ovarian tumors from January 2012 to December 2014 on a hospital information system—a picture archiving and communication system (PACS). We encountered 11 consecutive cases (median age, 42 years; range, 21–58 years) of metastatic ovarian tumors confirmed by pathology. Six gastric cancers, two colon cancers, one cervical cancer, one breast cancer and one thyroid cancer were found among the patients. It is obvious that the most common site of primary origin of metastatic ovarian cancers was stomach. Meanwhile, we detected 26 consecutive cases (median age, 56 years; range, 17–82 years) with pathologically confirmed primary epithelial ovarian cancers. None of these cases witnessed the history of malignant tumors except the present cancers. All the primary epithelial ovarian cancers had undergone surgery. Then the cancers were confirmed by histopathological pathologists in Shanghai First People’s Hospital. Consequently, ten serous cystadenocarcinoma, six clear cell cancers, five borderline malignancy, three mucinouscystadenocarcinoma and two endometrioid adenocarcinomas were found (Table 1).

**Table 1:** Summary of the cases

Tumor type		Total cases	Histopathologically confirmed cases
Metastatic ovarian tumors	Gastric cancer	6	6
	Colon cancer	2	2
	Thyroid cancer	1	1
	Uterine cervical cancer	1	1
	Breast cancer	1	1
	Subtotal	11	11
Primary ovarian tumors	Serous cystadenocarcinoma	10	10
	Clear cell carcinoma	6	6
	Borderline malignancy	5	5
	Mucinous cystadenocarcinoma	3	3
	Endometrioid adenocarcinoma	2	2
	Subtotal	26	26
Total		37	37

### MRI scanning

MRI examinations were performed on a 3T system (Signa HD, General Electric Healthcare, Milwaukee, WI, USA). The scan scope was from the umbilicus to the pubic symphysis in the caudocranial direction. For too large lesions to be totally scanned on axial imaging, a sagittal scanning sequence was adopted to contain as much of the total lesion as possible. First, routine MRI protocols were performed for the detection of the lesions, which contained fast spin-echo (FSE) T1-weighted images (T1WI), sagittal FSE T2-weighted images (T2WI) and fat-suppressed T2WI (FS T2WI) on the axial imaging. A diffusion weighted imaging (DWI)-MRI sequence contained an echo-planar imaging sequence with an array spatial-sensitivity-encoding technique (ASSET). The settings of the T1WI MRI protocol were: repetition time (TR) = 540 ms; echo time (TE) = 7 ms; number of excitations (NEX) = 2 and thickness = 7 mm. The parameters of the T2WI MRI protocol were: TR = 2400 ms; TE = 85 ms; NEX = 1 and thickness = 7 mm. The settings of the FS T2WI MRI protocol were: TR = 3800 ms; TE = 90 ms; NEX = 2 and thickness = 7 mm. The settings of the DWI MRI protocol were: TR = 4300 ms; TE = 63 ms; NEX = 6; thickness = 7 mm and the b value = 0 or 800 s/mm^2^. Second,a liver acquisition with a volume acceleration (LAVA) sequence was adopted for contrast-enhanced pelvic imaging, and a power injector (Missouri Ulrich, Ulm, Germany) was used to inject the contrast medium (Magnevist, Bayer Schering Pharma AG, Germany). The parameters of the LAVA MRI protocol were: TR = 3.5 ms; TE = 1.6 ms; NEX = 1; flip angle = 15°; band width =125 kHz and thickness = 2 mm. Subsequently, the images were obtained in multiple phases of contrast agent enhancement among the sagittal and axial planes—precontrast sagittal and axial oblique, postcontrast at 20 s, 40 s, 60 s, 80 s in the axial plane, and 120 s in the sagittal plane. The details of the scanning parameters of imaging are presented in Table 2.

**Table 2:** Details of parameters for MRI scanning protocols

Parameters	FSE-T1WI	FSE-T2WI	FS T2WI	EPI-DWI	LAVA
Repetition/echo time (ms)	540/7	2400/85	3800/90	4300/63	3.5/1.6
NEX	2	1	2	6	1
Thickness(mm)	7	7	7	7	2
Field of view (mm)	36	36	36	40	38
Matrix	320 × 224	256 × 224	256 × 224	96 × 130	320 × 224
Flip angle (degrees)					15

### MRI image analysis

The following lesion parameters were evaluated: 1) the maximum diameter of lesions; 2) laterality; 3) uniformity of locules (uniform or not uniform); 4) configuration: cystic-solid (less than half of solid component), solid (more than half of solid component); 5) DWI signal of solid components (moderate or high); 6) enhancement of solid portions (moderate or prominent enhancement, referring to the enhancement of myometrium); 7) ascites. The preoperative MRI diagnoses were correlated with histopathological results.

### Statistical analyses

Statistical analyses were performed with SPSS 19.0 for Windows (SPSS, Chicago, IL). The differences between metastatic ovarian tumors and primary epithelial ovarian cancers in laterality, configuration, uniformity of locules, DWI signal of solid components, and enhancement of solid portions were compared by Fisher’s exact test, Median age of patients, the maximum diameter of lesions and biomarkers (CA125, human epididymis protein 4 (HE4)) were compared by the Mann-Whitney test. The sensitivity, specificity, accuracy, positive predictive value (PPV), and negative predictive value (NPV) of the significant MRI features of metastatic ovarian tumors were calculated. *P* < 0.05 was considered statistically significant.

## Results

**Fig. 1:**
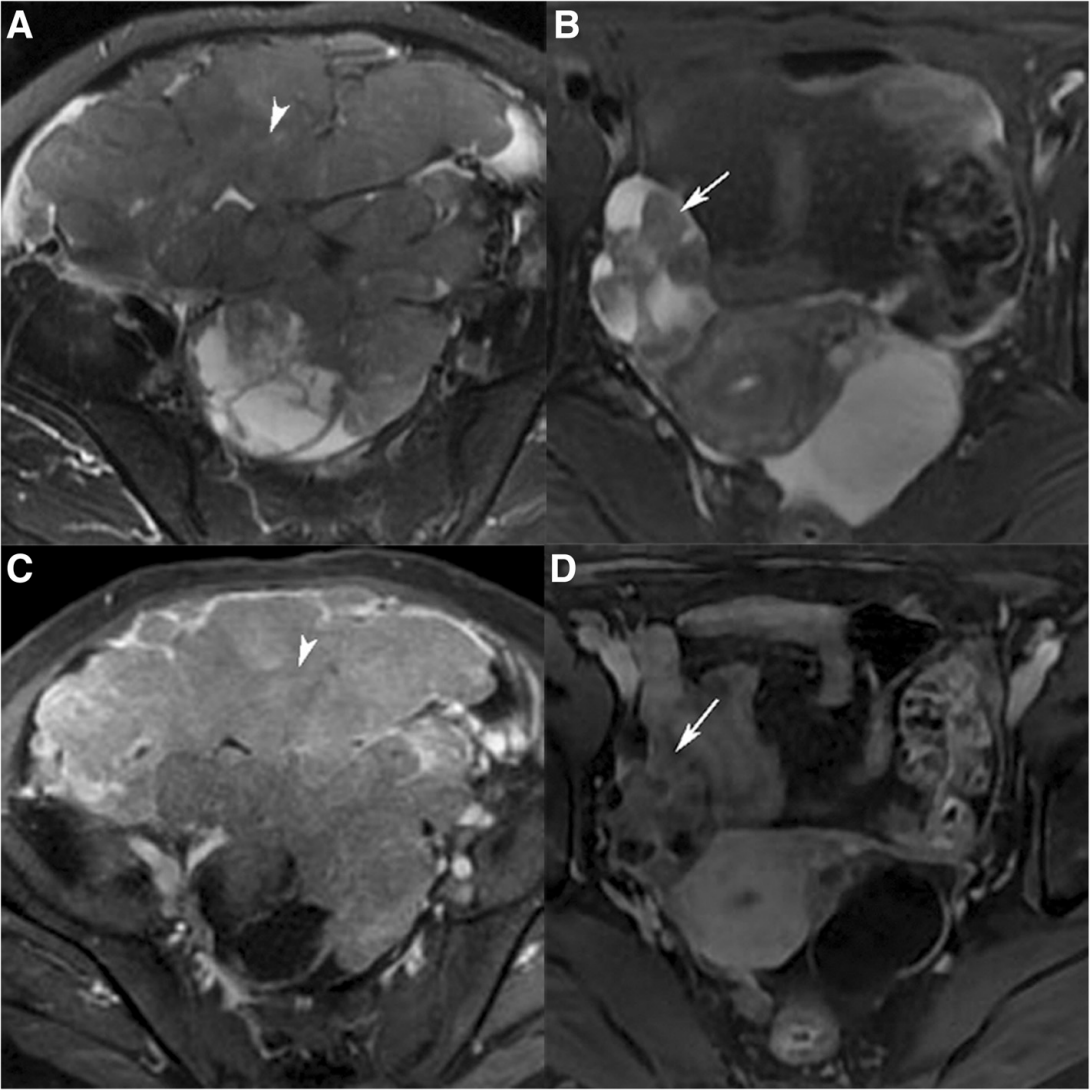
**a** and **c** A 58-year-old woman with serous cystadenocarcinoma on the right ovary. **a** Axial T2WI with fat saturation image showed the locules were not uniform. **c** Axial LAVA dynamic contrast-enhanced MRI image showed the solid portion was prominent enhancement (arrowhead). **b** and **d**: A 49-year-old woman with metastatic ovarian tumors on the right ovary. **b** Axial T2WI with fat saturation image showed the locules were uniform. **d** Axial LAVA dynamic contrast-enhanced MRI image showed the solid portion was moderate enhancement (arrow)

Metastatic ovarian tumors and primary epithelial ovarian cancers were found in the median patients who were 42 and 56 years old respectively (*P* = 0.015). Totally, seventeen tumors were found in 11 patients with metastatic ovarian tumors—bilateral tumors in six patients and unilateral tumors in five patients; thirty-one tumors were found in 26 patients with primary epithelial ovarian cancers—bilateral tumors in five patients and unilateral tumors in 21 patients (*P* = 0.032). The maximum tumor diameter was generated from 25 to 200 mm (median, 67 mm) in metastatic ovarian tumors versus 16 to 285 mm (median, 122 mm) in primary epithelial ovarian cancers (*P* = 0.005). The locules were uniform in 59 % of metastatic ovarian tumors versus 26 % of primary epithelial ovarian cancers (*P* = 0.024). The enhancement of solid portions was moderate in 76 % of metastatic ovarian tumors versus 45 % in primary epithelial ovarian cancers (*P* = 0.037). Figure 1 showed that the locules of a serous cystadenocarcinoma on the right ovary were not uniform on FS T2WI and the solid portion was prominent enhancement on LAVA dynamic contrast-enhanced MRI (DCE-MRI) (A and C). In contrast, the locules of a metastatic ovarian tumors on the right ovary were uniform on FS T2WI and the solid portion was moderate enhancement on LAVA DCE-MRI (B and D). CA125 and HE4 of metastatic ovarian tumors and primary epithelial ovarian cancers were significantly different (*P* = 0.033, 0.006, respectively). In all, there was a statistically significant difference in the median age of patients, the maximum diameter of lesions, laterality, uniformity of locules, enhancement of solid components, CA125 and HE4 between metastatic ovarian tumors and primary epithelial ovarian cancers. There was no statistically significant difference between the two groups in terms of configuration, DWI signal of solid components and ascites (*P* = 0.272, 0.428 and 0.108, respectively). The MRI features of metastatic ovarian tumors compared with primary epithelial ovarian cancers are shown in Table 3.

**Table 3:** Comparison of each parameter between metastatic and primary ovarian tumors

		Metastatic	Primary	*P* value
Patients’ age (years)	Median	42	56	0.015
Laterality	Unilateral cases	5	21	0.032
	Bilateral cases	6	5	
Maximum diameter of lesions (mm)	Median	67	122	0.005
Uniformity of locules	Uniform	10	8	0.024
	Not uniform	7	23	
Configuration	Cystic-solid	10	23	0.272
	Solid	7	8	
DWI signal of solid components	Intermediate	5	6	0.428
	High	12	25	
Enhancement of solid portions	Moderate	13	14	0.037
	Prominent	4	17	
CA125	Elevated cases	3	17	0.033
HE4	Elevated cases	1	15	0.006
Ascites		5	19	0.108

Diagnostic parameters for the characterization of the metastatic ovarian tumors are listed in Table 4. The combination of ovarian lesions with any one of the following three features, patients’ age, small size, and bilaterality, yielded sensitivity, specificity, accuracy, PPV, and NPV for identifying metastatic ovarian tumors of 100, 87, 92, 89, and 100 %, respectively.

**Table 4:** Accuracy of MRI in characterizing ovarian lesions as metastatic ovarian tumors

MRI features	Sensitivity (%)	Specificity (%)	Accuracy (%)	PPV (%)	NPV (%)
Patients’ age(<50 years)	65 (11/17)	81 (25/31)	75 (36/48)	65 (11/17)	81 (25/31)
Small size (<100 mm)	82 (14/17)	61 (19/31)	69 (33/48)	54 (14/26)	86 (19/22)
Bilaterality	71 (12/17)	68 (21/31)	69 (33/48)	55 (12/22)	81 (21/26)
Uniformity of locules	59 (10/17)	74 (23/31)	69 (33/48)	50 (10/20)	77 (23/30)
Moderate enhancement	76 (13/17)	55 (17/31)	63(30/48)	48 (13/27)	81 (17/21)

*PPV* positive predictive value, *NPV* negative predictive value

## Discussion

It is important to discriminate between metastatic ovarian tumors and primary ovarian cancers to select the most appropriate management which influences the prognosis. However, it is difficult to distinguish those two groups of tumors because they both show the imaging features of malignant tumors. There are some studies concerning the imaging features of metastatic ovarian tumors. Metastatic ovarian tumors and primary epithelial ovarian cancers are difficult to distinguish by CT as they both display mixed cystic and solid lesions [6]. Bilaterality has been reported as a specific feature of metastatic ovarian tumors [7]. However, La Fianza et al concluded that bilaterality was not significantly different between secondary and primary ovarian cancers after reviewing more than eighty cases of ovarian tumors [8]. In our study, 54.5 % of metastatic tumors showed bilaterality in contrast to 23.8 % of primary epithelial ovarian cancers (*P* = 0.032), roughly in agreement with Kim et al. [7]. Therefore, bilaterality still seems to be an important factor in diagnosing metastatic ovarian tumors by imaging.

Khunamornpong et al reported that the maximum diameter of unilateral ovarian carcinomas less than 100 mm were considered as metastases, and more than 100 mm as primary ovarian cancers [4]. Jung et al advocated that a cutoff of 150 mm for classifying unilateral tumors resulted in a higher diagnostic accuracy [5]. In our study, the median maximum tumor diameter is 101 mm in unilateral metastatic ovarian tumors versus 154 mm in primary epithelial ovarian cancers. Perhaps, the maximum diameter of tumors ranged from100 to 150 mm may be an overlap area between unilateral metastatic ovarian tumors and primary ovarian cancers.

Tanaka et al. reported that metastatic ovarian tumors tended to be composed of uniform cysts compared with primary mucinous tumors [2]. Our results suggested that metastatic ovarian tumors tend to be composed of uniform locules in contrast to primary epithelial ovarian cancers, which is similar to Tanaka et al. [2]. Histopathological findings show that metastatic tumors proliferate in a more uniform manner than primary ovarian cancers [2]. We think that features such as mucus productivity of tumor cells may be more uniform in metastatic tumors. Consequently, this could explain that the size of locules with metastatic ovarian tumors would be more uniform than those of primary epithelial ovarian cancers.

Primary ovarian neoplasms often appeared as a prominent enhancement [9–12]. Our study was also in accordance with previously reported studies. Our study advocated that metastatic ovarian tumors often appeared as a moderate enhancement versus primary epithelial ovarian cancers with a prominent enhancement.

DWI-MRI evaluation of ovarian tumors has yet been reported as a useful tool for differentiating malignant tumors from benign ovarian lesions [13–18]. However, there is no helpful role for distinguishing two types of malignant tumors by DWI-MRI. Moreover, there was no statistically significant difference between the two groups in terms of DWI signal of solid components. Besides, there was also no statistically significant difference between the two groups in terms of presence of ascites.

For other features, the patients of metastatic ovarian tumors were younger than those of primary epithelial ovarian cancers. This is possibly due to the younger population of some kinds of cancers recently. In one series of gastric cancers in young women (age < 36 years), 55 % had ovarian involvement [19]. Thus, in a woman with a known gastric carcinoma, the development of bilateral ovarian masses on imaging will be considered as highly likely to be secondary metastases rather than primary ovarian cancers. CA125 and HE4 are significantly higher in primary ovarian cancers than metastatic ovarian tumors. But, the level of CA125 and HE4 are not correlated to the size of the lesions, which is corresponded by Liao et al. [20].

There were some limitations in our study. First, a limited number of patients were studied. Therefore, more cases are needed to further investigate the value of these imaging features for diagnosing metastatic ovarian tumors. Second, the interreader variability was not evaluated. Third, a selection bias was inevitably appeared as the retrospective inherent nature of the study.

## Conclusions

In conclusion, metastatic ovarian tumors seem to be smaller in size, more bilateral, more uniform in locules and more moderate enhancement in solid portions than those of primary ovarian cancers. Metastatic ovarian tumors were with less elevated levels of CA125 and HE4 in contrast with primary ovarian cancers. Although the sensitivity, specificity, and accuracy of any features are not sufficient for diagnoses, the combination of three key features (patients’ age, small size, and bilaterality) tends to have a high sensitivity, specificity, and accuracy for identifying metastatic ovarian tumors. The radiologist must depend on a combination of the imaging features as well as the clinical examinations in order to diagnose metastatic ovarian tumors.
